# Supplementary material for: Multi-modal nonlinear optical and thermal imaging platform for label-free characterization of biological tissue
Source: Sci Rep. 2021 Apr 13;11:8067. doi: 10.1038/s41598-021-86774-2 (PMC8044215; doi:10.1038/s41598-021-86774-2)
Supplement: Supplementary file 1 — Supplementary Information 1. [file 41598_2021_86774_MOESM1_ESM.pdf]

Title

Multi-modal Nonlinear Optical and Thermal Imaging Platform for Label-Free Characterization of Biological Tissue

Authors

Wilson R Adams,[1] Brian Mehl,[2] Eric Lieser,[2] Manqing Wang,[3] Shane Patton,[2] Graham A Throckmorton,[1] J Logan Jenkins,[1] Jeremy B Ford,[1] Rekha Gautam,[1] Jeff Brooker,[2] E. Duco Jansen,[1,4] Anita Mahadevan-Jansen[1,4,*]

Affiliations

[1]Vanderbilt University, Dept of Biomedical Engineering, Nashville, TN, 37235, USA.

[2]Thorlabs Imaging Research, Sterling, VA, USA.

[3]Chongqing University, College of Bioengineering, Chongqing, China.

[4]Vanderbilt University Medical Center, Dept of Neurosurgery, Nashville, TN, 37232, USA

*anita.mahadevan-jansen@vanderbilt.edu

Supplemental Materials

Table S1: Summary of emission filters and excitation wavelengths used for multimodal imaging. All filters obtained from Semrock (Brattleboro, VT, USA).

Table S2: Summary of measured system resolutions across multiple imaging modalities.

Table S3: Detailed summary of point spread function calculations for nonlinear imaging modalities.

Figure S1: Sample Lateral Resolution Characterization for SHG imaging in a porcine mitral valve sample. Gaussian fitting performed in FIJI.

Figure S2: Signal to Noise (SNR) and signal to background (SBR) calculation with a Vegetable Oil meniscus at 2927cm-1 in Fiji. SNR was calculated to be 34.6 (SNR = Mean 1 / StdDev 2). SBR was calculated to be 2.30 (SBR = Mean 1 / Mean 2).

Figure S3: To-scale representation of the overlap of multimodal imaging fields of view for the 3 imaging arms of MANTIS.

Figure S4: Thermal images of cultured 3T3 Fibroblasts. A) Cells imaged without any aqueous medium. B) Image of aqueous medium front advancing over cells. The absorption of water in the short-wave infrared is high, making cell culture medium difficult to image through with blackbody thermal contrast.

Figure S5: Porcine Mitral Valve imaging with endogenous autofluorescence from elastin (left) and collagen SHG (right). Image width is 520-µm.

Figure S6: Simultaneous SHG (cyan) and SRS (2880-cm-1, lipid dominant resonance, orange) imaging of an unstained murine cervix unfixed frozen section. The stroma (dense in collagen) and epithelium are easily discernable based on relative concentrations on ratio of SHG and SRS signals. Blood vessels lamina propria are also visible with SHG contrast.

Figure S7: Composite multimode images of an *ex vivo* rat sciatic nerve. (A) CARS signal at 2927cm^{-1} (B, green), SRS signal at 2927cm^{-1} (C, red), SHG signal (D, cyan), and multiphoton fluorescence of FluoroMyelin Green (E, Grey). All scale bars are 100um.

Figure S8: Ex vivo rat sciatic nerve samples imaged with multimodal nonlinear imaging from Supp. Fig. S7. Two different modalities can be combined in different ways to visualize tissue structure.

Figure S9: Supplementary Figure 9: Ex vivo rat sciatic nerve samples imaged with SRS (red, myelin at 2927cm-1) and SHG (cyan, collagen). Rescaled SHG images are shown to highlight intrafasicular collagen, which is in lower abundance and overall signal then epineurial collagen.

Supplementary Tables

Table S1: Summary of emission filters and excitation wavelengths used for multimodal imaging. All filters obtained from Semrock (Brattleboro, VT, USA).

Contrast	Filter (Center/Passband FWHM)	Excitation Wavelengths
CH-band CARS	625nm/90nm	792-803nm & 1040nm
SRS	890nm/310nm	792-803nm & 1040nm
NADH/Blue Fluorescence	460nm/30nm	780nm
FAD/Green Fluorescence	525nm/30nm	934nm
SHG	460nm/30nm	900nm
Propidium Iodide / Orange Fluorescence	625nm/90nm	1040nm

Table S2: Summary of measured system resolutions across multiple imaging modalities.

Modality	Lateral Resolution (μm)	Axial Resolution (μm)
CARS	0.632	3.009
SRS	0.833	3.136
MPF	0.359	1.502
SHG	0.388	X
Thermal	6.9	X

Table S3: Detailed summary of point spread function calculations for nonlinear imaging modalities.

CARS						
					Mean	STD
**x**	0.646	0.615	0.636	**x**	**0.6323**	0.0158
**y**	0.773	0.69	0.772	**y**	**0.7450**	0.0476
**z**	2.758	3.282	2.986	**z**	**3.0087**	0.2627
SRS						
**x**	0.849	0.743	0.906	**x**	**0.8327**	0.0827
**y**	0.896	0.793	0.86	**y**	**0.8497**	0.0523
**z**	3.36	2.813	3.235	**z**	**3.1360**	0.2866
MPF						
**x**	0.379	0.272	0.426	**x**	**0.3590**	0.0789
**y**	0.286	0.413	0.377	**y**	**0.3587**	0.0655
**z**	1.473	1.432	1.601	**z**	**1.5020**	0.0882
SHG						
**L**	0.388	0.383	0.433	**L**	**0.4013**	0.0275

Supplementary Figures:

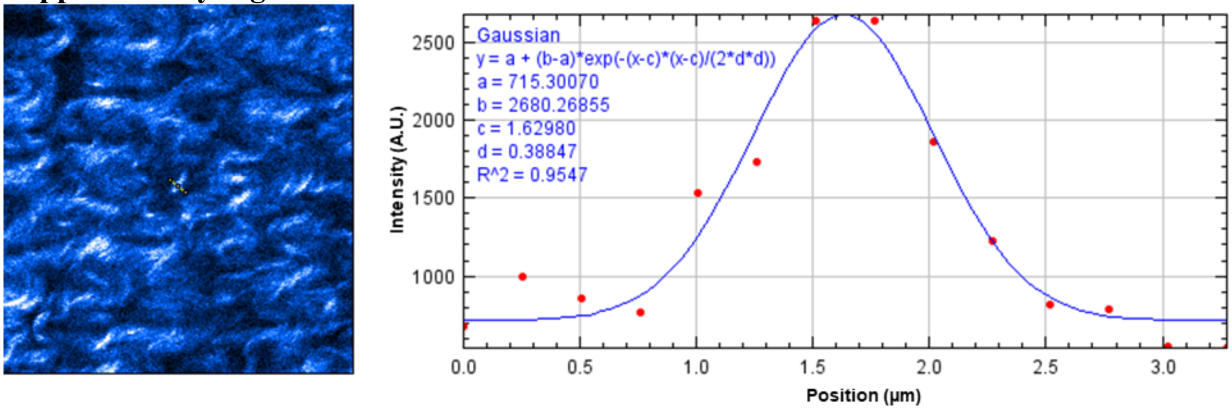

Figure S1: Sample Lateral Resolution Characterization for SHG imaging in a porcine mitral valve sample. Gaussian fitting performed in FIJI.

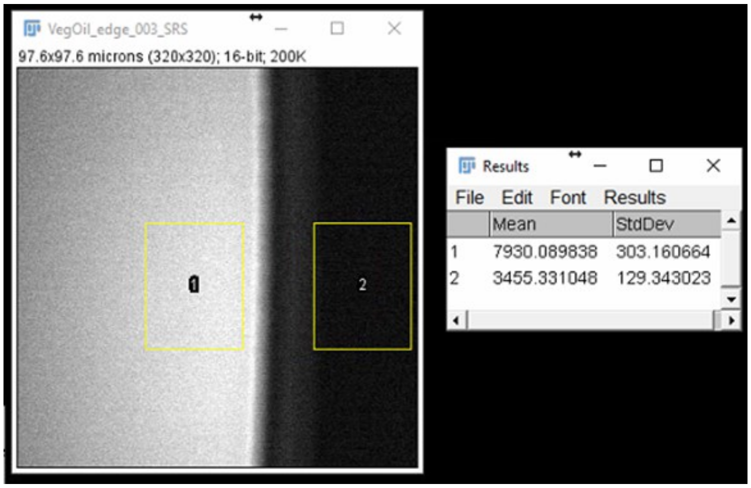

Figure S2: Signal to Noise (SNR) and signal to background (SBR) calculation with a Vegetable Oil meniscus at 2927cm-1 in Fiji. SNR was calculated to be 34.6 (SNR = Mean 1 / StdDev 2). SBR was calculated to be 2.30 (SBR = Mean 1 / Mean 2).

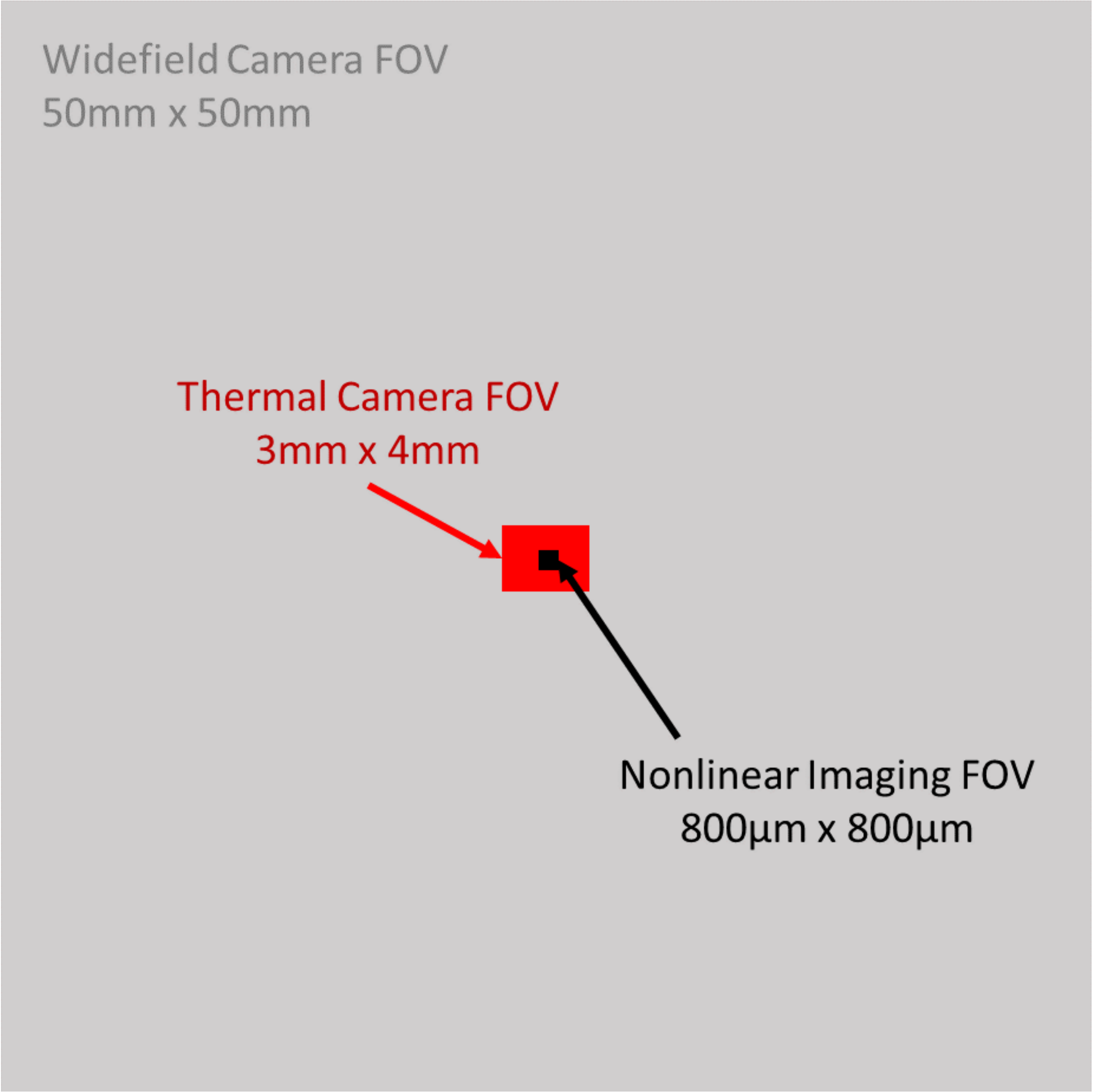

Figure S3: To-scale representation of the overlap of multimodal imaging fields of view for the 3 imaging arms of MANTIS.

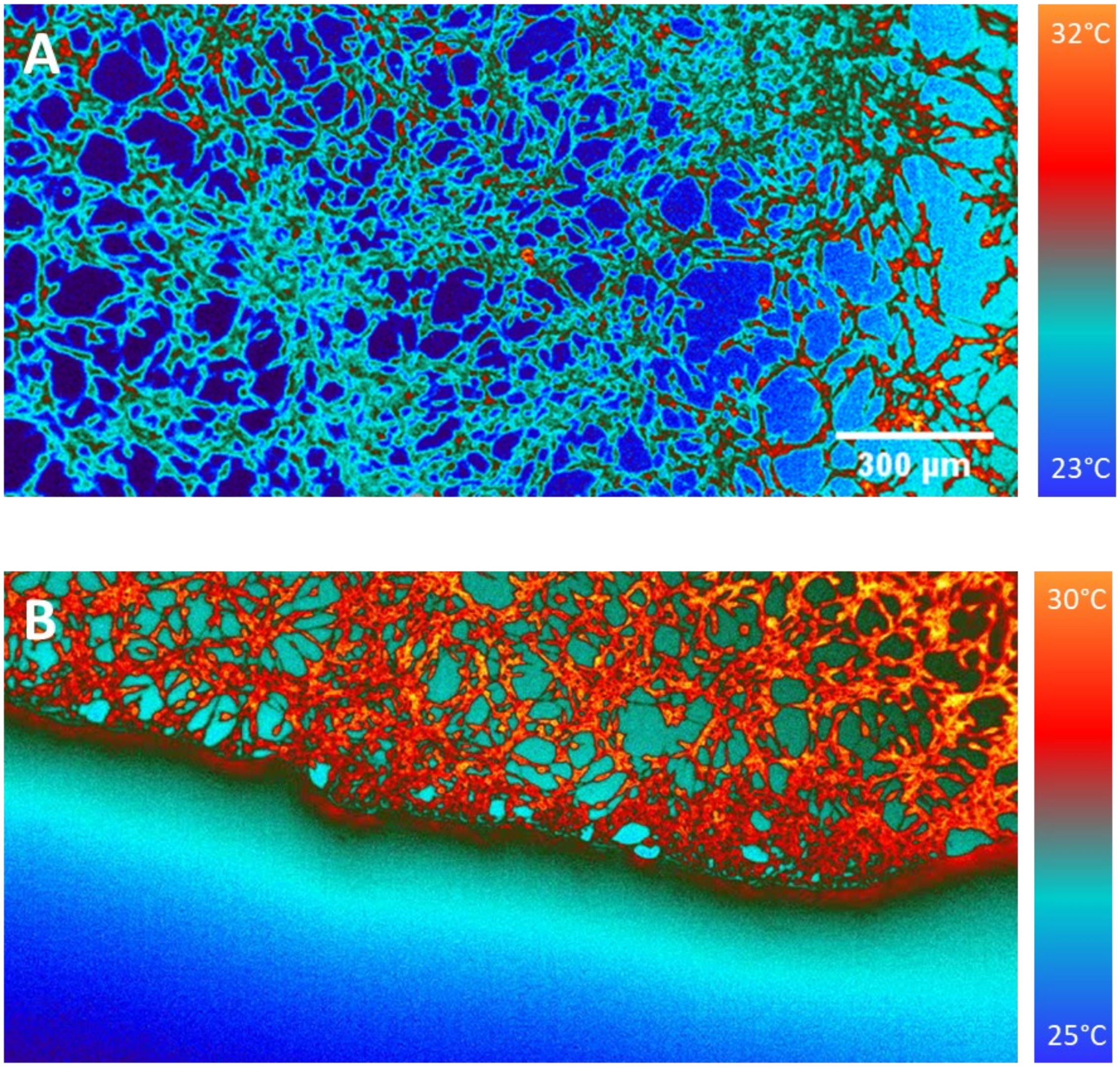

Figure S4: Thermal images of cultured 3T3 Fibroblasts. A) Cells imaged without any aqueous medium. B) Image of aqueous medium front advancing over cells. The absorption of water in the short-wave infrared is high, making cell culture medium difficult to image through with blackbody thermal contrast.

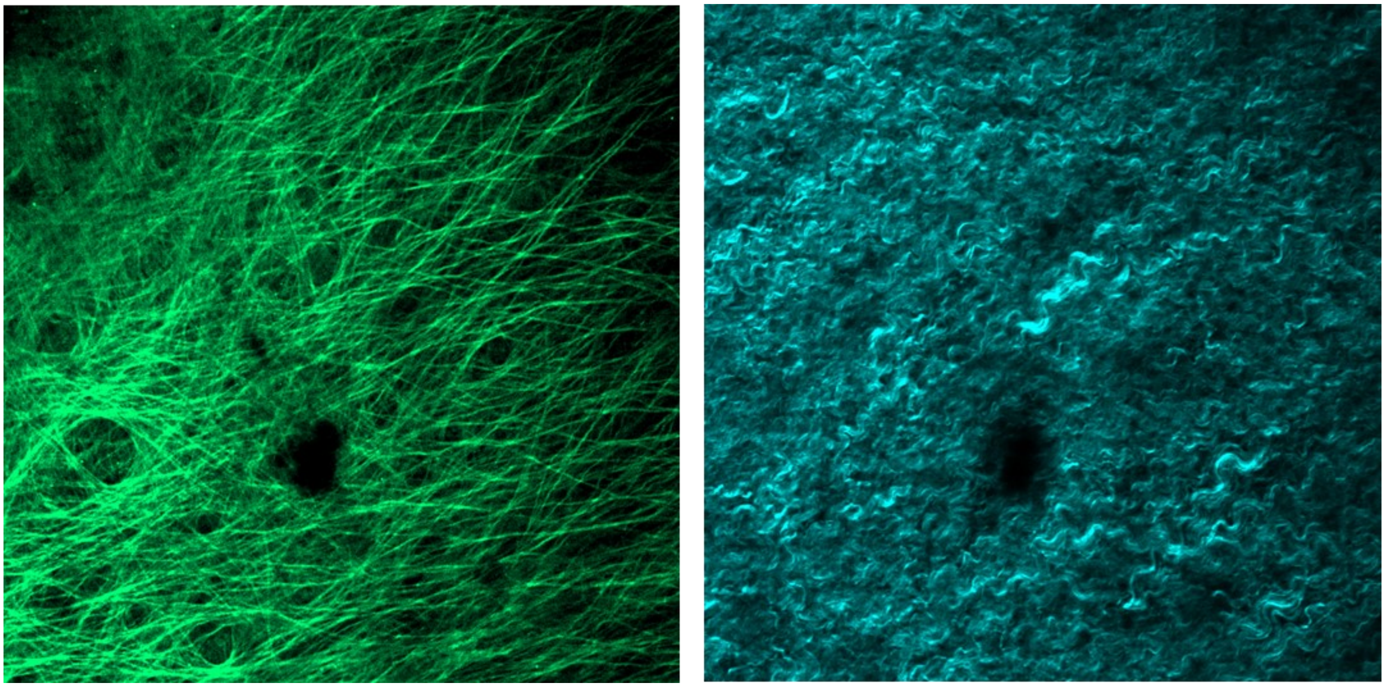

Figure S5: Porcine Mitral Valve imaging with endogenous autofluorescence from elastin (left) and collagen SHG (right). Image width is 520-µm.

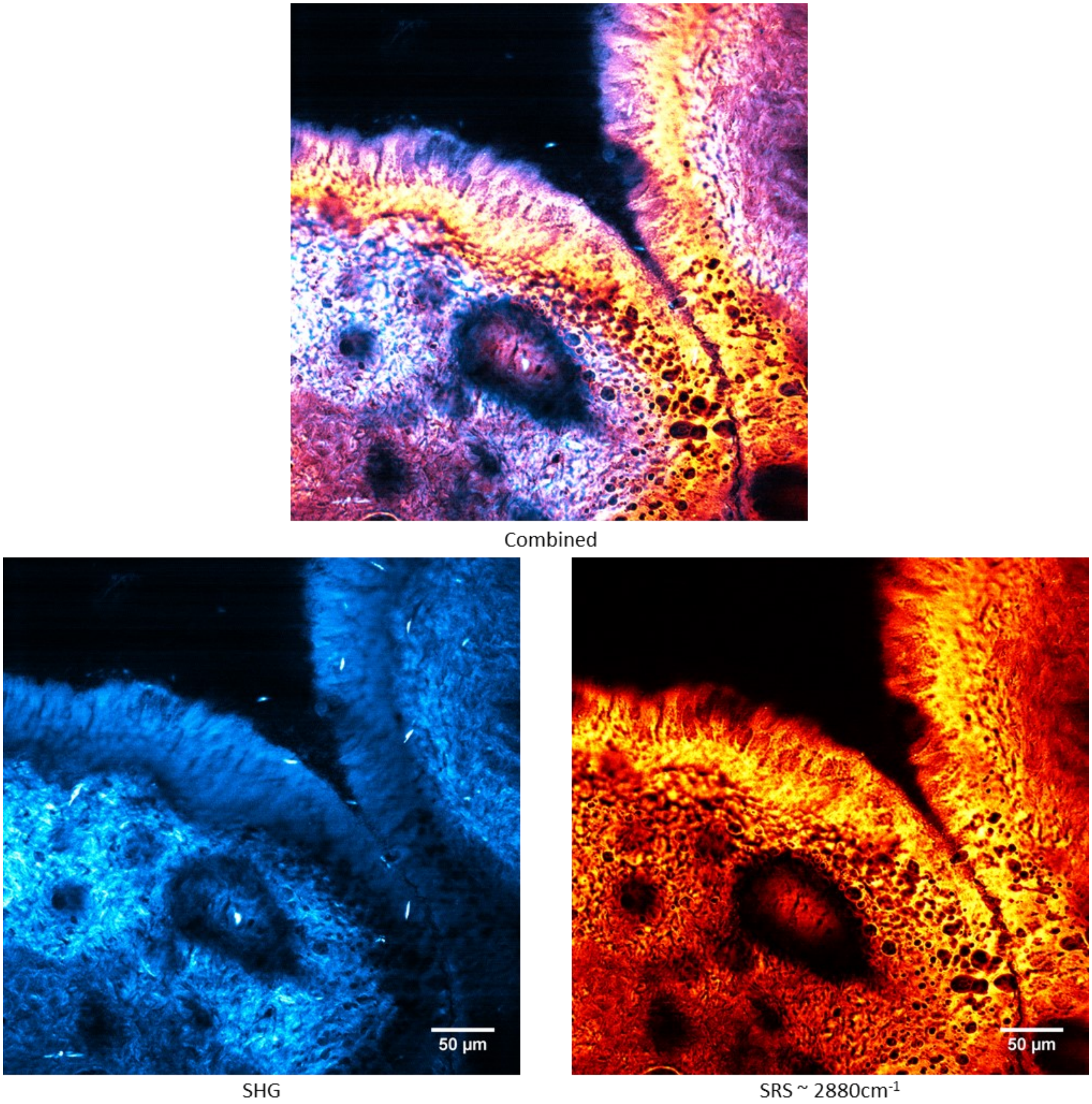

Figure S6: Simultaneous SHG (cyan) and SRS (2880-cm-1, lipid dominant resonance, orange) imaging of an unstained murine cervix unfixed frozen section. The stroma (dense in collagen) and epithelium are easily discernable based on relative concentrations on ratio of SHG and SRS signals. Blood vessels lamina propria are also visible with SHG contrast.

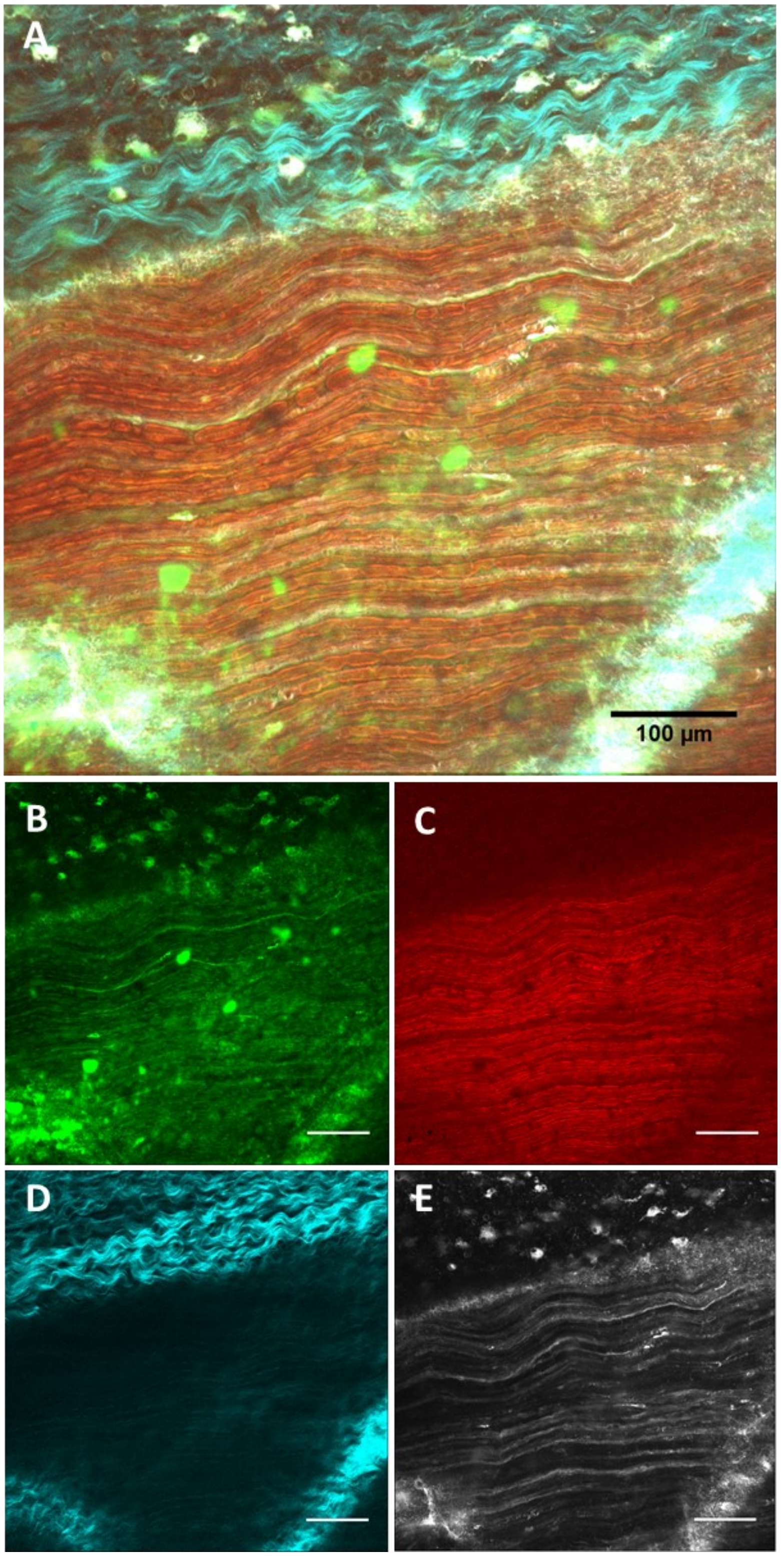

Figure S7: Composite multimode image of an *ex vivo* rat sciatic nerve. (A) CARS signal at 2927cm-1 (B, green), SRS signal at 2927cm-1 (C, red), SHG signal (D, cyan), and multiphoton fluorescence of FluoroMyelin Green (E, Grey). All scale bars are 100um.

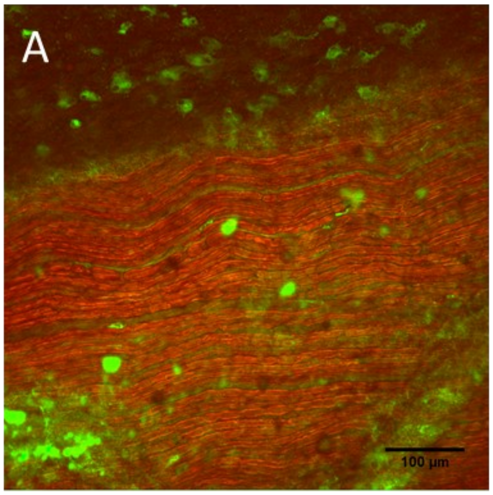

CARS (green) |SRS at 2927-cm^{-1} (red)

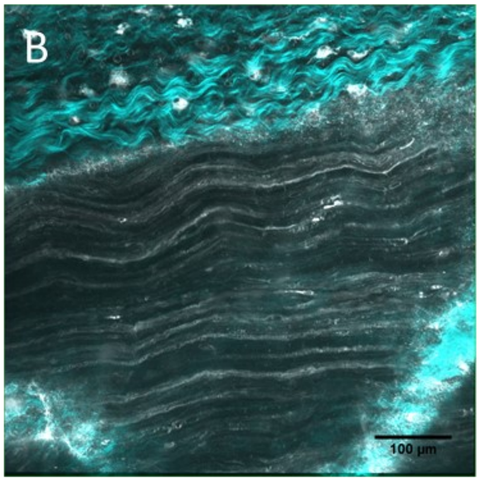

SHG (cyan) | Multiphoton Fluorescence of FluoroMyelin Green (grey)

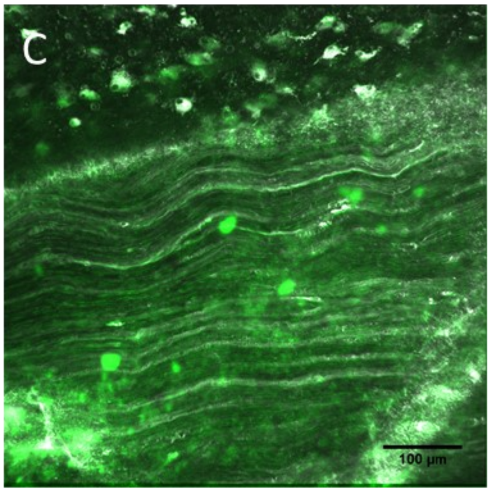

CARS at 2927-cm^{-1} (green) | Multiphoton Fluorescence of FluoroMyelin Green (grey)

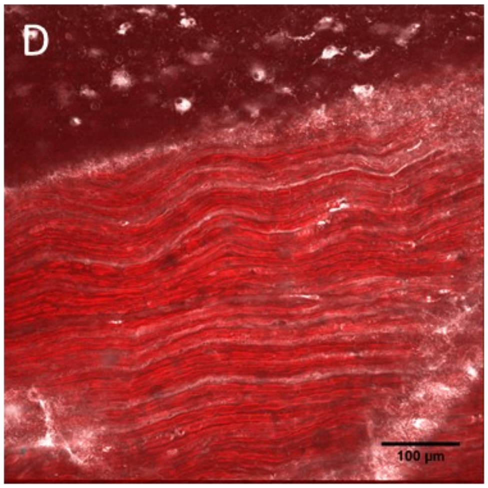

SRS at 2927-cm^{-1} (red) | Multiphoton Fluorescence of FluoroMyelin Green (grey)

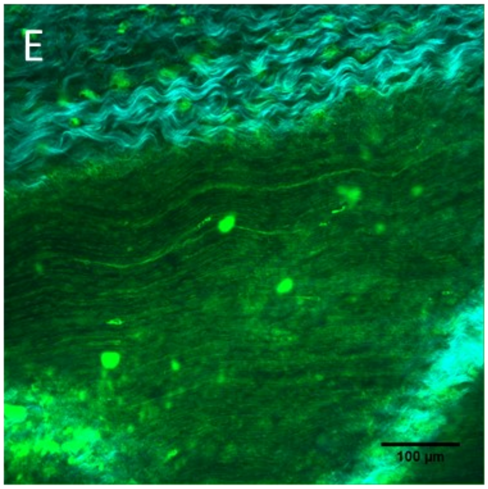

CARS at 2927-cm^{-1} (greed) |.SHG (cyan)

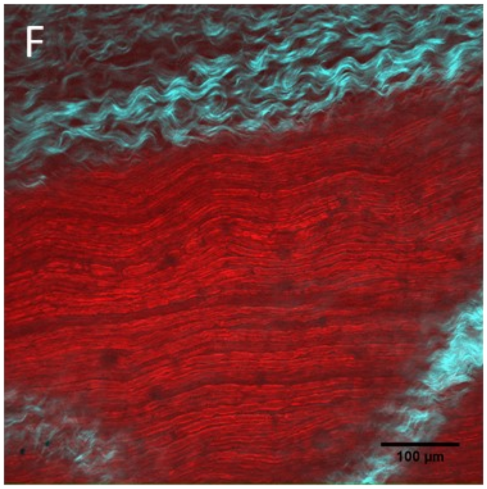

SRS at 2927-cm^{-1} (red) | SHG (cyan)

Figure S8: Ex vivo rat sciatic nerve samples imaged with multimodal nonlinear imaging from Supp. Fig. 7. (A-F) Two different modalities can be combined in different ways to visualize tissue structure.

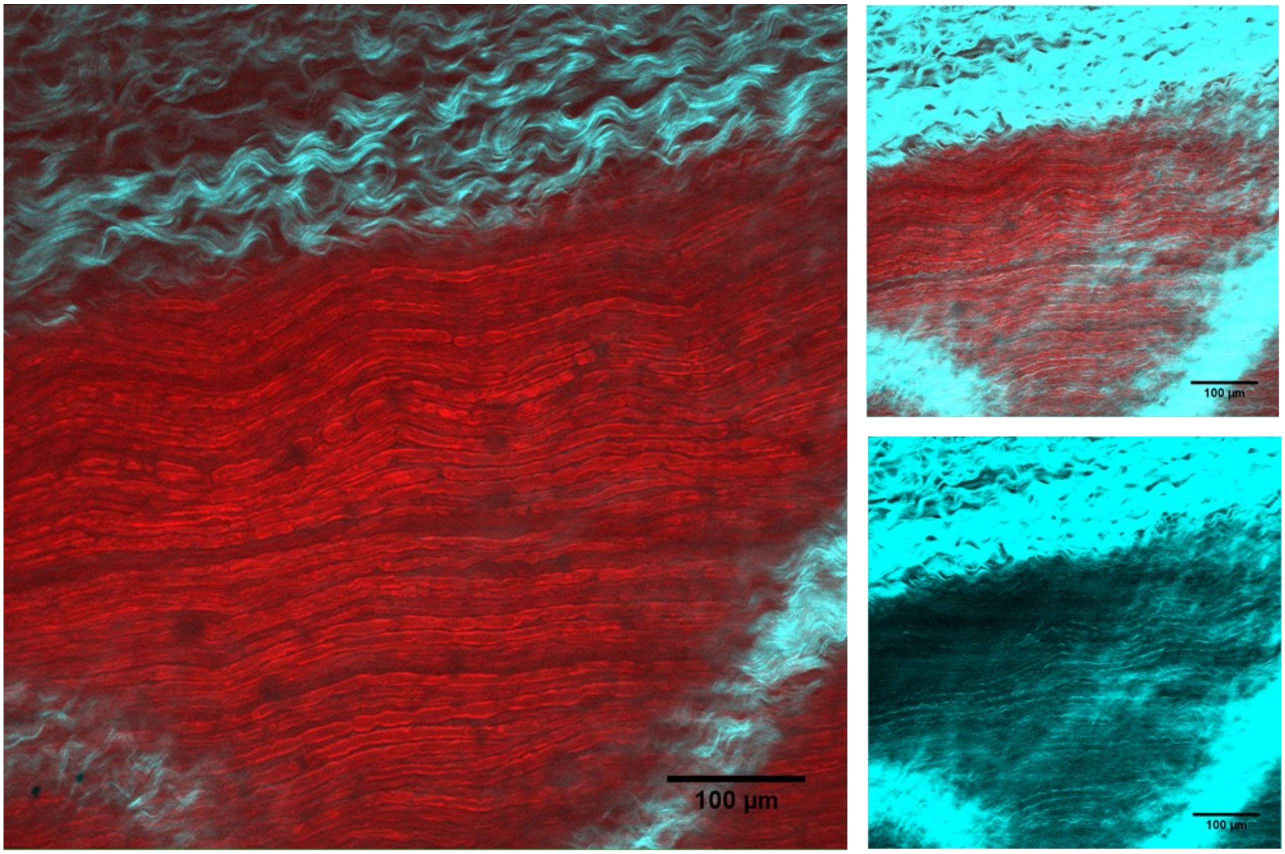

SRS at 2927cm^{-1} vs. SHG

SHG contrast adjusted to show intrafasicular collagen structures

Figure S9: Ex vivo rat sciatic nerve samples imaged with SRS (red, myelin at 2927cm-1) and SHG (cyan, collagen). Rescaled SHG images are shown to highlight intrafasicular collagen, which is in lower abundance and overall signal then epineurial collagen.
